# Characteristics of and Public Health Responses to the Coronavirus Disease 2019 Outbreak in China

**DOI:** 10.3390/jcm9020575

**Published:** 2020-02-20

**Authors:** Sheng-Qun Deng, Hong-Juan Peng

**Affiliations:** Department of Pathogen Biology, Guangdong Provincial Key Laboratory of Tropical Disease Research, School of Public Health, Southern Medical University, Guangzhou 510515, China; dengshengqun@163.com

**Keywords:** coronavirus, pneumonia, 2019-nCoV, clinical characteristics, diagnosis, prevention and control, treatment, public health, COVID-19, SARS-CoV-2

## Abstract

In December 2019, cases of unidentified pneumonia with a history of exposure in the Huanan Seafood Market were reported in Wuhan, Hubei Province. A novel coronavirus, SARS-CoV-2, was identified to be accountable for this disease. Human-to-human transmission is confirmed, and this disease (named COVID-19 by World Health Organization (WHO)) spread rapidly around the country and the world. As of 18 February 2020, the number of confirmed cases had reached 75,199 with 2009 fatalities. The COVID-19 resulted in a much lower case-fatality rate (about 2.67%) among the confirmed cases, compared with Severe Acute Respiratory Syndrome (SARS) and Middle East Respiratory Syndrome (MERS). Among the symptom composition of the 45 fatality cases collected from the released official reports, the top four are fever, cough, short of breath, and chest tightness/pain. The major comorbidities of the fatality cases include hypertension, diabetes, coronary heart disease, cerebral infarction, and chronic bronchitis. The source of the virus and the pathogenesis of this disease are still unconfirmed. No specific therapeutic drug has been found. The Chinese Government has initiated a level-1 public health response to prevent the spread of the disease. Meanwhile, it is also crucial to speed up the development of vaccines and drugs for treatment, which will enable us to defeat COVID-19 as soon as possible.

## 1. Introduction

In December 2019, a cluster of pneumonia of unknown etiology was detected in Wuhan City, Hubei Province of China. The first 27 reported cases were all related to Huanan Seafood Wholesale Market, which sells aquatic products, live poultries, and wild animals [1]. The first batch of cases identified later showed no exposure or even no relation to Huanan Wholesale Market, and the human-to-human transmission was confirmed; moreover, nosocomial infections were reported in some health care workers [2,3,4]. The Chinese Center for Disease Control and Prevention (CDC) and Chinese health authorities later identified and announced that a new coronavirus (2019-nCoV) was accountable for the outbreak of this pneumonia in Wuhan [5]. Thereafter, this disease was named Coronavirus Disease 2019 (COVID-19) by World Health Organization (WHO), and the causative virus was designated as SARS-CoV-2 by the International Committee on Taxonomy of Viruses [6]. Within one and a half months, as of midnight of 18 February 2020, the novel coronavirus pneumonia (COVID-19) had spread from Hubei to 34 provinces in China and another 25 countries, resulting in 75,199 confirmed cases with 2009 deaths (Table 1) [7]. At present, the number of cases is increasing rapidly in China and even around the world, which is a big threat to public health. Thirty-one provinces of China have initiated a level-1 public health response. The aim of this article is to provide a timely review of the characteristics of the COVID-19 outbreak including the epidemiology, pathogenicity, clinical features, and measures of treatment, prevention, and control for this disease.

## 2. The Epidemiology of COVID-19

On 31 December 2019, Wuhan Municipal Health Commission reported a number of unknown pneumonia cases related to Huanan Seafood Wholesale Market, 27 cases were hospitalized, seven of which were in serious condition [1]. On 5 February 2020, Wuhan Municipal Health Committee reported that 59 cases of viral pneumonia with unknown etiology were detected in Wuhan, including seven severe cases, but no clear evidence was found for “human-to-human” transmission [8]. On Jan 11, Wuhan Municipal Health Committee issued a new report confirming that the pathogen of the viral pneumonia of unknown cause was initially determined as a new coronavirus [9]. On 20 February 2020, it was officially confirmed that “human-to-human” transmission and nosocomial infection had occurred [2,3].

**Figure 1:**
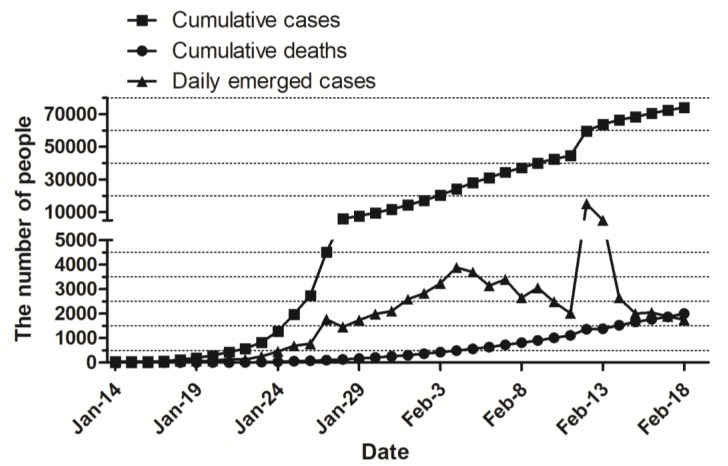
Daily cumulative/emerged number of confirmed cases and fatal cases of Coronavirus Disease 2019 (COVID-19) in Mainland China. As of 18 February 2020, the total number of confirmed cases and deaths reached 74,185 and 2004, respectively. Since 16 February 2020, the total number of confirmed cases increased quickly; the daily emerging cases increased steadily to 3886 on February 4, and then fluctuated to 2015 on 11 February 2020; the fatality cases number increased slowly to 2004 cases on 18 February 2020. The cumulative and daily emerged cases number jumped to 59,804 and 15,152, respectively, on 12 February 2020.

Since 16 February 2020, the cumulative COVID-19 case number increased quickly; meanwhile, the daily emerging case number increased steadily to 3886 on 4 February 2020, and then fluctuated to 2015 on 11 February 2020. The fatality cases number increased steadily to 2004 cases on 18 February 2020. The cumulative and daily emerged cases number jumped to 59,804 and 15,152, respectively, on 12 February 2020 (Figure 1). This fierce growth of cumulative and daily emerged cases number in one day is due to the improvement of diagnosis standard for confirmed cases in Hubei province, in which the suspected cases with pneumonia imaging characteristics are categorized as clinical diagnosis cases. As a result, the patients can receive standard treatment as soon as possible. All data are from the National Health Commission of the People’s Republic of China [10].

The COVID-19 resulted in much lower mortality (about 2.67% up-to-date) among the confirmed cases, compared with Severe Acute Respiratory Syndrome (SARS) at 9.60% (November 2002–July 2003) and Middle East Respiratory Syndrome (MERS) at 34.4% (April 2012–November 2019) (Table 1). The median ages for the patients of COVID-19, SARS, and MERS are 55.5, 41.3, and 52.8 years old, respectively. COVID-19 and MERS patients share similargender composition of females (32%) and males (67%), but SARS patients show almost the same proportion of males (46.9%) and females (53.1%).

According to the “Diagnosis &Treatment Scheme for Novel Corona Virus Pneumonia (Trial) 6th Edition”, the source of infection is majorly the COVID-19 patients, even the asymptomatic patients can also be the source of infection. The transmission way is majorly through respiratory droplets and contacting. People are generally susceptible to this virus.

## 3. Pathogenic Characteristics of Coronavirus

Coronavirus is a single strand positive RNA (+ssRNA) virus, belonging to order Nidovirales, family Coronaviridae, and subfamily Orthocoronavirinae [13]. According to the characteristics of serotype and genome, the coronavirus subfamily is divided into four genera: α, β, γ, and δ [14]. There are six kinds of coronaviruses known to infect humans, including 229E and NL63 of α genus [15,16], OC43, HKU1, Middle East respiratory syndrome-associated coronavirus (MERSr-CoV), and severe acute respiratory syndrome-associated coronavirus (SARSr-CoV) of β genus [16,17]. The coronavirus isolated from the lower respiratory tract of patients with unidentified pneumonia in Wuhan is a new type of coronavirus (SARS-CoV-2) belonging to genus β, and subgenus sarbe [5]. SARS-CoV-2 is different from the zoonotic MERSr-CoV and SARSr-CoV and becomes the seventh coronavirus to infect humans [5]. The phylogenetic analysis of the coronaviruses based on full-length genome sequences shows that SARS-CoV-2 has the smallest genetic distance from bat coronavirus, but only about 45%–90% similarity with SARSr-CoV, and a lower similarity of 20%–60% with MERSr-CoV [18]. Therefore, a bat is probably the original host of SARS-CoV-2, although the intermediate host may still exist in the process of transmission from bats to human beings.

Coronavirus has an envelope, the particles are round or oval, often pleomorphic, with a diameter of 50–200 nm [18]. S protein is located on the surface of the virus and forms a rod-shaped structure. As one of the main antigenic proteins of the virus, the S protein gene is the main target used for typing [19]. Xu et al. also reported that SARS-CoV-2 S-protein supports a strong interaction with human angiotensin-converting enzyme 2 (ACE2) molecules, which means that the virus poses a significant public health risk for human transmission by the S-protein–ACE2 binding pathway [18].

The knowledge of the physical and chemical characteristics of coronaviruses mostly comes from the study of SARS-CoV and MERS-CoV. The coronaviruses are sensitive to heat and can be killed at 56 °C for 30 min. In addition, ether, 75% ethanol, chlorine disinfectant, peracetic acid, and chloroform can effectively inactivate the virus, but not chlorhexidine [20].

## 4. Clinical Characteristics of COVID-19

### 4.1. Clinical Manifestations

According to the “Diagnosis & Treatment Scheme for Novel Coronavirus Pneumonia (Trial) 6th Edition” enacted by the National Health Commission of the People’s Republic of China on 19 February 2020, the incubation time after exposure is about 1–14 days [20]. Fever, fatigue, and a dry cough are the main manifestations. Nasal obstruction, runny nose, and other upper respiratory symptoms are rare. About half of the patients developed dyspnea one week later, and severe cases developed rapidly into acute respiratory distress syndrome, septic shock, hard-to-correct metabolic acidosis, and coagulation dysfunction. Severe and critical patients may present moderate to low fever, or even no obvious fever. Some patients have mild onset symptoms, no fever, and mostly recovered after one week. Most patients have a favorable prognosis, although some patients are left in a critical condition, or do not survive. The aged patients and the patients with basic diseases have worse prognosis. Children cases are relatively mild.

### 4.2. Laboratory Examination

In the early stages of the disease, the total number of leukocytes in peripheral blood is normal or decreased, the lymphocyte count is decreased, and some patients present elevated levels of liver enzyme, muscle enzyme, and myoglobin; some severe cases present elevated troponin level. Most patients show elevated C-reactive protein (CRP) and erythrocyte sedimentation rate (ESR), and normal procalcitonin. In severe cases, the patients present with increased D-dimer and progressively decreased peripheral blood lymphocyte [20]. Compared with non-ICU patients, the plasma levels of IL2, IL7, IL10, GSCF, IP10, MCP1, MIP1a, and TNF-α were higher in ICU patients [21].

### 4.3. Chest Imaging

All patients suffered from pneumonia, and chest CT scans showed shadows in the lung [20]. In the early stages of the disease, patients present with multiple small patch shadows and interstitial changes, especially in the extrapulmonary zone. It further progresses to multiple ground glass shadows and infiltrative shadows in both lungs. In severe cases, lung consolidation may occur, but pleural effusion is rare [20].

**Figure 2:**
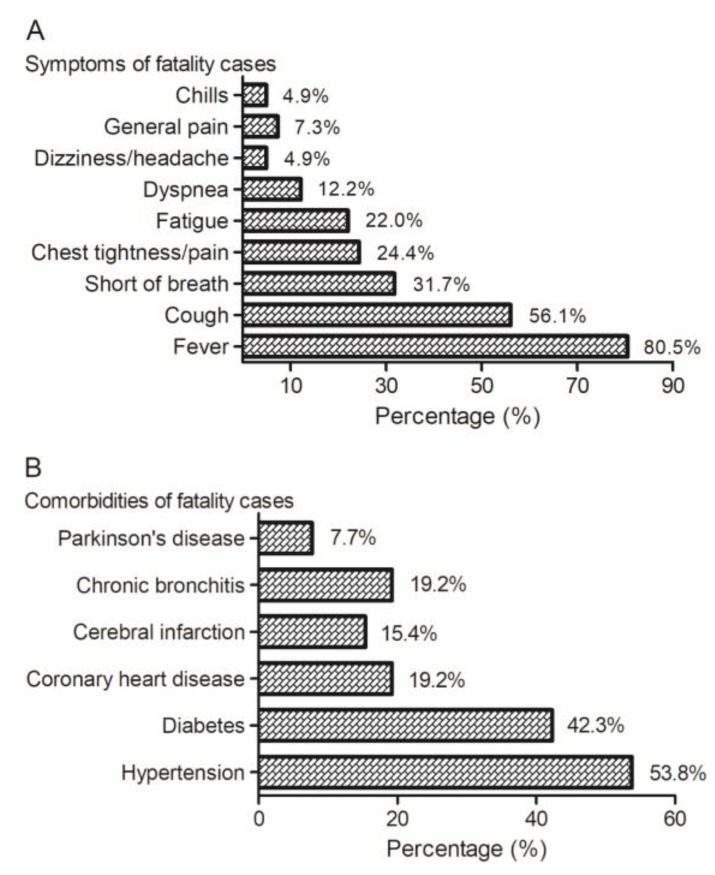
Clinical symptoms and comorbidities of the patients died of COVID-19. (**A**) Among 41 cases with fatalities, the symptoms include fever (80.5%), cough (56.1%), shortness of breath (31.7%), chest tightness/pain (24.4%), fatigue (22.0%), dyspnea (12.2%), dizziness/headache (4.9%), general pain (7.3%), and chills (4.9%). (**B**) Among 26 cases with fatalities, the major comorbidities are hypertension (53.8%), diabetes (42.3%), coronary heart disease (19.2%), cerebral infarction (15.4%), chronic bronchitis (19.2%), and Parkinson’s disease (7.7%). The case information is from reports released by different provincial Health Commissions, including the National Health Commission (the links are shown in Supplementary Data 1).

To provide more information about the disease for the treatment in clinics, we collected detailed information about the fatal cases released by official channels. A total of 41 fatal cases were used to reveal the symptoms of the deaths, showing the symptom composition of fever (80.5%), cough (56.1%), short of breath (31.7%), chest tightness/pain (24.4%), fatigue (22.0%), dyspnea (12.2%), dizziness/headache (4.9%), general pain (7.3%) and chills (4.9%) (Figure 2). A total of 26 cases with fatalities were used to disclose the dangerous comorbidities, showing that the major comorbidities are hypertension (53.8%), diabetes (42.3%), coronary heart disease (19.2%), cerebral infarction (15.4%), chronic bronchitis (19.2%) and Parkinson’s disease (7.7%) (Figure 2). Chen N et al. reported that, among 99 confirmed cases, the common symptoms are fever (83%), cough (82%), bilateral pneumonia (75%), short of breath (31%), muscle ache (11%), confusion (9%), headache (8%), sore throat (5%), rhinorrhoea (4%), chest pain (2%), diarrhoea (2%), and nausea and vomiting (1%) [4]. Huang C et al. reported that, among 41 confirmed cases, the symptom composition is as follows: pneumonia (100%), fever (98%), cough (76%), lymphopenia (63%), Dyspnea (55%), fatigue (44%), sputum production (28%), headache (8%), hemoptysis (5%), and diarrhea (3%); and the underlying diseases included diabetes (20%), hypertension (15%), and cardiovascular disease (15%) [21]. Though the symptom compositions reported for the confirmed cases are similar to that of the fatality cases we collected, the percentages of hypertension, diabetes, and coronary heart diseases are much higher among the fatality cases than among the confirmed cases. This may indicate that the comorbidities probably are important factors resulted in death of COVID-19 patients.

## 5. Diagnosis of COVID-19

The diagnosis was based on a set of clinical criteria recommended by the National Health Commission of the People’s Republic of China and the National Administration of Traditional Chinese Medicine [20].

### 5.1. Suspected Cases

The cases comply with any item of A and any two items of B, or with 3 items of B as follows. (A) Epidemiological history: within two weeks before disease onset, 1. have a history of travel or residence in the district with case report; 2. have contacted the patients positive with nucleic acid detection; 3. have contacted with the patients with fever and respiratory symptoms from the district with case report; 4. disease onset in clustering. (B) Clinical manifestations: 1. Have fever and/or respiratory tract symptoms; 2. Have pneumonia with image characteristics mentioned above; 3. In early stage of the disease, have normal or decreased total number of leukocytes, or decreased lymphocyte count.

### 5.2. Confirmed Cases

The unconfirmed cases met the criteria of the suspected cases and are identified positive with SARS-CoV-2 RNA, by real-time RT-PCR or gene sequencing, from the sputum, throat swab, lower respiratory tract secretion, or other samples collected from patients.

### 5.3. Clinical Typing of the Confirmed Cases

Mild cases: have mild symptoms, no pneumonia manifestation in chest image;

Common cases: have fever, respiratory symptoms, and pneumonia manifestation in chest image;

Severe cases: comply with any item of the follows. (A) dyspnea, respiratory rate ≥30 times/min; (B) at resting state, finger oxygen saturation ≤ 93%; (C) PaO2/FiO2 ≤ 300 mmHg (1mmHg = 0.133 kPa, PaO_2_: arterial partial pressure of oxygen, FiO_2_: fractional concentration of inspired oxygen);

Critical cases: comply with any item of the follows. (A) show respiratory failure and mechanical ventilation is required; (B) present with shock; (C) combine with other organ failure needing Intensive Care Unit (ICU) monitoring and treatment; (D) chest imaging shows multilobe lesions or progress of lesion focus within 48 h ≥ 50%; (E) combine with other clinical conditions requiring hospitalization.

## 6. Treatment of COVID-19

### 6.1. Treatment Area Decision According to the Disease Severity

Suspected and confirmed cases should be treated in isolation in hospitals with effective isolation and protective conditions. The suspected cases should be isolated in a single room, and the confirmed cases can be accepted in the same room. Critical cases should be treated in ICU as soon as possible.

### 6.2. General Treatment

- Bed rest, strengthen supportive treatment, ensure sufficient energy; pay attention to water-electrolytes balance and maintain the stability of the internal environment; closely monitor vital signs and finger oxygen saturation, and so on.
- Monitor the blood routine, urine routine, C-reactive protein (CRP) and health indications (liver enzyme, myocardial enzyme, renal function, etc.), coagulation function, arterial blood gas analysis if necessary, and recheck chest imaging.
- According to the change of oxygen saturation, give effective oxygen therapy in time, including oxygen given by nasal catheter or mask. If necessary, apply high flow oxygen therapy via the nose, noninvasive or invasive mechanical ventilation, and so on.
- Antiviral treatment: no effective antiviral drug at present. Treat with IFN-α aerosol inhalation (five million U per time for adults, two times per day), and/or Lopinavir/Ritonavir oral administration (two tablets per time, two times per day).
- Antibiotic treatment: avoid blind and improper use of antibiotics, especially the combination use of broad-spectrum antibiotics. Strengthen bacteriological monitoring. Antibiotics should be used in time in secondary bacterial infection.

### 6.3. Treatment of Severe and Critical Cases

- Treatment principle: based on symptomatic treatment, actively prevent and treat complications, treat basic diseases, prevent secondary infection, and timely apply organ function support.
- Respiratory support: apply noninvasive mechanical ventilation for two hours, if the condition is not improved, or the patient is intolerable to noninvasive ventilation, accompanied with increased airway secretions, severe coughing, or unstable hemodynamics, the patient should be transferred to invasive mechanical ventilation in time. The “lung-protective ventilation strategy” with low tidal volume should be adopted in invasive mechanical ventilation to reduce ventilator-associated lung injury. If necessary, ventilation in the prone position, recruitment maneuver, or extracorporeal membrane oxygenation (ECMO) can be used.
- Circulation support: improve microcirculation based on full fluid resuscitation, use vasoactive drugs, and apply hemodynamic monitoring if necessary.
- Others: according to the degree of dyspnea and the progress of chest imaging, use glucocorticoids appropriately for a short time (3–5 days) with the recommended dose no more than what is equivalent to methylprednisolone 1–2 mg/kg·day.

## 7. Prevention and Control

As of 26 January 2020, 30 provinces have initiated a level-1 public health response to control COVID-19 [22]. A level-1 response means that during the occurrence of a particularly serious public health emergency, the provincial headquarters shall organize and coordinate the emergency response work within its administrative area according to the decision deployment and unified command of the State Council [22]. Fever observation rooms shall be set up at stations, airports, ports, and so on to detect the body temperature of passengers entering and leaving the area and implement observation/registration for the suspicious patients. The government under its jurisdiction shall, in accordance with the law, take compulsory measures to restrict all kinds of the congregation, and ensure the supply of living resources. They will also ensure the sufficient supply of masks, disinfectants, and other protective articles on the market, and standardize the market order. The strengthening of public health surveillance, hygiene knowledge publicity, and monitoring of public places and key groups is required. Comprehensive medical institutions and some specialized hospitals should be prepared to accept COVID-19 patients to ensure that severe and critical cases can be differentiated, diagnosed, and effectively treated in time. The health administration departments, public health departments, and medical institutions at all (province, city, county, district, township, and street) levels, and social organizations shall function in epidemic prevention and control and provide guidance for patients and close contact families for disease prevention [23].

The responsibilities of the organizations are shown in Table 2. In accordance with the working principle of “prevention first, prevention and control combined, scientific guidance and timely treatment”, the prevention and control work shall be carried out in a coordinated and standardized way [23].

## 8. Discussion

The recent outbreak of the unknown severe pneumonia in China is caused by a novel coronavirus named 2019-nCoV [2], later was designated SARS-CoV-2 by the International Committee on Taxonomy of Viruses. This virus and the SARSr-CoV/MERSr-CoV share a common ancestor [2]. Compared with SARSr-CoV and MERSr-CoV, SARS-CoV-2 results in much lower mortality in patients but has a comparable infection ability. From the analysis of the fatal cases of this novel coronavirus pneumonia, the comorbidities of hypertension, diabetes, coronary heart disease, cerebral infarction, and chronic bronchitis were found to be dangerous factors that resulted in death.

In 2002 and 2003, the outbreak of SARS brought a disaster to the people of the world, especially the Chinese people [24,25]. Fortunately, SARS was finally defeated, and Chinese health departments upgraded their disease prevention and control system by summing up their experiences of fighting SARS. Thus, when COVID-19 appeared, the whole country quickly entered a state of fighting against the new infectious disease. Policies led by the National Health Commission have been formulated and implemented efficiently, and Chinese scientists identified the etiology of the disease in no more than a month. However, new cases are increasing every day, showing a trend of spreading to the whole country and across the world.

COVID-19 appeared just one month before the Spring Festival of China, and the massive population flow has brought great challenges for disease prevention and control. This virus can be transmitted from human to human and no effective treatment drug has been found. The most effective prevention and control measures are to find suspected patients and close contacts, confirm patients and virus carriers, and block the transmission through isolation, disinfection, and personal protection. Therefore, early detection, isolation, and treatment of patients are the key measures to control the source of infection and reduce the infection rate. It is also crucial to avoid nosocomial infection by strengthening the management of medical staff and patients. Health education on knowledge for disease prevention and control is also important. Finally, if we want to eliminate the threat of this novel coronavirus pneumonia similar to SARS, we need to learn more about the pathogenesis of the virus and develop specific vaccines and therapeutic drugs as soon as possible.

## Figures and Tables

**Table 1:** The key information about the coronavirus infection outbreaks.

Coronavirus Infection Outbreak	Total Confirm Cases		Median Age (Years Old)	Number of Deaths	Case-Fatality Rate (%)	Countries Reported Cases	References
	Female (%)	Male (%)					
COVID-19 (November 2019–18 February 2020)	75,199		55.5 *	2009	2.67 11.0 *	26	[10] [4]
	32 *	67 *					
SARS (November 2002–July 2003)	8098		41.3	774	9.60	26	WHO [11]
	53.1	46.9					
MERS (April 2012–November 2019)	2494		52.8	858	34.4	27	WHO [12]
	26.3	73.7					

COVID-19: coronavirus disease 2019; SARS: severe acute respiratory syndrome; MERS: Middle East respiratory syndrome; WHO, World Health Organization. * Data are from reference [4].

**Table 2:** Responsibilities for the different organizations at all (province, city, county, district, township, and street) levels in the outbreak of COVID-19.

Organization at all Levels	Health Administration Department	Center for Diseases Control	Medical Institutions
**Objectives**	To timely find and report the **COVID-19** cases, understand the disease characteristics and possible sources of infection, standardize the management of close contacts, and prevent the spread of the epidemic.		
**Responsibilities**	Overall guidance of epidemic control, organizing a technical expert group for prevention and control; formulation and improvement of relevant work and technical schemes, and implementation of funds and materials for disease prevention and control; tracking and management of close contacts.	Organization, coordination, supervision, and evaluation of the monitoring work; collection, analysis, report, and feedback of the monitoring data; epidemiological investigation; strengthening laboratory testing ability, bio-safety protection awareness, and technical training; carrying out health education and publicity and risk communication to the public.	Case detection and report, isolation, diagnosis, and treatment; clinical management and prevention and control of nosocomial infections; sample collection and detection, and training of medical staff in the institution.

## Supplementary Materials

The following are available online at https://www.mdpi.com/2077-0383/9/2/575/s1, Supplementary Data 1, The case information is from the reports released by official channels.
