# Supplementary material for: Characteristics of and Public Health Responses to the Coronavirus Disease 2019 Outbreak in China
Source: J Clin Med. 2020 Feb 20;9(2):575. doi: 10.3390/jcm9020575 (PMC7074453; doi:10.3390/jcm9020575)
Supplement: Supplementary file 1 [file jcm-09-00575-s001.pdf]

**Supplementary Data 1**

The case information is from the reports released by official channels (the links are as fllows).

## Fatality Cases

link accessed on Feb 3, 2020

Patient 1-17: http://www.nhc.gov.cn/yjb/s3578/202001/5d19a4f6d3154b9fae328918ed2e3c8a.shtml.

Patient 18-32:http://wjw.hubei.gov.cn/bmdt/ztzl/fkxxgzbdgrfyyq/xxfb/202001/t20200125_2014855.shtml.

Patient 33-39: http://wjw.hubei.gov.cn/fbjd/dtyw/202001/t20200124_2014626.shtml.

Patient 40 : http://www.hebwsjs.gov.cn/index.do?id=395652&templet=content&cid=3714.

Patient 41 :http://wjw.beijing.gov.cn/xwzx_20031/wnxw/202001/t20200127_1621454.html.

Patient 42: http://wsjkw.sh.gov.cn/xwfb/20200126/734a56a6c9684cebbac859e6fcb9a97c.html.

Patient 43: http://wst.hainan.gov.cn/swjw/rdzt/yqfk/202001/t20200127_2741242.html.

Patient 43-45: https://baijiahao.baidu.com/s?id=1657283292685706125&wfr=spider&for=pc.
